# Application of Ozonation-Biodegradation Hybrid System for Polycyclic Aromatic Hydrocarbons Degradation

**DOI:** 10.3390/ijerph20075347

**Published:** 2023-03-31

**Authors:** Magdalena Olak-Kucharczyk, Natalia Festinger, Wojciech Smułek

**Affiliations:** 1 Łukasiewicz Research Network—Lodz Institute of Technology, Maria Skłodowska-Curie 19/27, 90-570 Lodz, Poland; 2 Institute of Chemical Technology and Engineering, Poznan University of Technology, Berdychowo 4, 60-695 Poznan, Poland

**Keywords:** PAHs biodegradation, creosote hydrocarbons degradation, creosote hydrocarbons model wastewater, ozonation-biodegradation hybrid system, pre-ozonation, microbial activity

## Abstract

Creosote, a mixture of polycyclic aromatic hydrocarbons (PAHs), was and is a wood impregnate of widespread use. Over the years the accumulation of creosote PAHs in soils and freshwaters has increased, causing a threat to ecosystems. The combined ozonation-biodegradation process is proposed to improve the slow and inefficient biodegradation of creosote hydrocarbons. The impact of different ozonation methods on the biodegradation of model wastewater was evaluated. The biodegradation rate, the changes in chemical oxygen demand, and the total organic carbon concentration were measured in order to provide insight into the process. Moreover, the bacteria consortium activity was monitored during the biodegradation step of the process. The collected data confirmed the research hypothesis, which was that the hybrid method can improve biodegradation. The pre-ozonation followed by inoculation with a bacteria consortium resulted in a significant increase in the biodegradation rate. It allows for the shortening of the time required for the consortium to reach maximum degradation effectiveness and cell activity. Hence, the study gives an important and useful perspective for the decontamination of creosote-polluted ecosystems.

## 1. Introduction

Increasing industrialization and negative anthropogenic activities have caused severe environmental pollution worldwide [1]. Although efforts to reduce emissions of toxic organic compounds into the environment have been ongoing for decades, their negative impact on the environment and on societies remains significant. In many cases these compounds self-degrade very slowly in the environment so that their concentrations in contaminated sites remain high for a very long time [2]. Polycyclic aromatic hydrocarbons (PAHs), as widespread environmental pollutants, are generally characterized by high melting and boiling points, low solubility, and low vapor pressure [3], and have carcinogenic, mutagenic, teratogenic, and estrogenic properties and can pose a significant threat to human health [4]. PAHs are formed as a product of incomplete combustion in various combustion sources (coal, oil, wood, and automobile emissions) [3,5,6]. Due to their high lipophilicity and stability, PAHs accumulate in the fatty tissues of fish after food ingestion or through sorption by the gills and skin [7]. Creosote, a mixture of hydrocarbons, in particular PAHs, has been used for many decades in many countries around the world to preserve wooden products, including fences, posts, masts, farm buildings, etc. [8,9]. This makes the sources of creosote contamination widespread. The use of these treated materials leads to the progressive release of creosote into the soil, surface water, and wastewater. The most common biological methods for the treatment of PAHs in water are phytoremediation and microbial bioremediation [3]. For sustainable environmental cleanup, bioremediation is widely preferred because it is considered to be an effective, economical, and environmentally friendly method. Microorganisms used in bioremediation detoxify (by degradation, mineralization, and accumulation) many harmful and biodegradable pollutants, which they convert into less harmful forms [1]. The activity of microorganisms is influenced by their species, their genes, and the conditions of the study. Due to the harmful effects of PAHs on some microbes, microorganisms must adapt to the prevailing conditions and form new microbial communities. Strains selected from contaminated areas show a high capacity to degrade PAHs [4]. They can be multiplied and transferred to other contaminated areas. In addition, the DNA of such microorganisms contains resistance or degradation genes that can be isolated and recombined to increase the efficiency of bioremediation [10]. Both PAHs and their metabolites can affect the degradation of PAHs. For example, through co-metabolism, microorganisms can utilize poorly available PAHs in the presence of readily available PAHs as carbon and energy sources [4].

The biodegradation of creosote, like any PAH mixture, is hampered by the relatively small number of microorganisms capable of this process [11]. It is usually necessary to use bioaugmentation, i.e., to inoculate the contaminated area or water with consortia specialised strains with high biodegradation potential [9,12]. Biodegradation of PAHs can be assisted by various methods. These include combined stimulation with sodium acetate/phthalic acid [13], biosurfactants for oil spills [14], interactions with biocarbon [1] and co-composting with animal manures [3]. Another solution is to use physicochemical methods aimed at pre-oxidation or the decomposition of PAHs [15].

One of the effective chemical methods for many micropollutants including pharmaceuticals and pesticides [16,17], preservative agents [18], dyes [19] as well as PAHs [20,21] is ozonation. It is well known that ozone is a strong oxidant that can react with chemical compounds directly (molecular ozone as a main oxidant) and indirectly (hydroxyl radicals generated via ozone decomposition are main oxidants). It should be noted that ozone is a selective oxidant and primarily attacks chemicals that are electronically dense, e.g., containing an aromatic ring, while hydroxyl radicals are non-selective species. Ozonation has been applied for PAHs removal from different media, including water, soil, as well as waste-activated sludge [20,21,22,23]. Ji and coworkers studied the ozonation of petroleum hydrocarbons in seawater [22]. They observed the rapid oxidation of PAHs at various gaseous ozone concentrations and the acceleration of their degradation in the presence of the oil dispersant and with increasing salinity. Important roles in the degradation process are played by both direct and indirect ozonation [22].

Ozone-based processes can be applied in combination with other methods such as biodegradation [24,25,26], which allows for the reduction in the cost of degradation. The literature illustrates different configurations of ozonation and biological degradation, such as ozonation-biodegradation, biodegradation-ozonation, and biodegradation-ozonation-biodegradation sequences [24,25,27,28]. During the pre-ozonation process, soluble and biodegradable compounds to support microbial growth and activity can be formed, therefore ozone pre-treatment usually enhances the biodegradability of wastewater [29]. Furthermore, biodegradation can be applied as a polishing step of the treatment [25]. On the other hand, the post-ozonation process can lead to the removal of resistant micropollutant residuals. The different combination of ozonation and biological methods were applied for detoxification of industrial textile wastewater [24], oil sands process water [28], pharmaceutical wastewater [30,31], olive mill wastewater [32], and urban wastewater [33]. The literature includes examples of the application of ozonation combined with the biodegradation process for PAHs removal [23,34,35,36]. For example, Kulik et al. studied the removal of PAHs from creosote contaminated sand and peat [37]. To the best of our knowledge, the topic of the combined ozonation and biodegradation of PAHs using microbial strains isolated from environmental samples has not been significantly investigated in recent years.

The aim of this study was to check the degradation of creosote hydrocarbons through the utilization of the combined treatment process: ozonation and biodegradation. Ozonation was applied as the first step. As a biodegradation culture, the consortium of five microbial strains isolated from hydrocarbon-contaminated environmental samples were used. The influence of ozone concentration and the procedure of culture preparation on the removal of PAHs were investigated.

## 2. Materials and Methods

### 2.1. Materials

#### 2.1.1. Chemicals

All chemicals used in the research were of analytical grade and were purchased from Merck (Darmstadt, Germany). The nutrient broth was purchased from BTL Sp. z o.o. (Łódź, Poland). The creosote (type B) was purchased from Centrala Obrotu Towarami Masowymi DAW-BYTOM Sp. z o.o. (Bytom, Poland).

#### 2.1.2. Bacterial Strains

The biodegradation cultures were inoculated with the consortium of five microbial strains isolated from hydrocarbon-contaminated environmental samples: *Pseudomonas* sp. MChB (GenBank No. KU563540), *Pseudomonas* sp. OS4 (GenBank No. KP096512), *Raoultella planticola* SA2 (GenBank No. KP096517), *Achromobacter* sp. KW1 (GenBank No. KP096519), and *Rahnella aquatilis* DA2 (GenBank No. KP096518).

### 2.2. Methods

#### 2.2.1. Culture Preparation

The microorganisms’ cultures were prepared according to the procedure described by Smułek et al. [6] with some modifications. Briefly, bacterial strains were revived in nutrient broth (a portion of biomass per loop in 20 mL of broth), incubated for 48 h at 30 °C, and then the cultures were centrifuged and suspended in saline to give OD_600nm_ of 0.9–1.0. After equilibration of OD_600nm_, suspensions of strains were mixed in a ratio of 1:1:1:1:1 *v*/*v*. We added the inoculum to the finished cultures at a ratio of 5 mL of suspension per 100 mL of culture. The synthetic wastewater was prepared according to OECD (The Organisation for Economic Co-operation and Development) procedure: “Test No. 303: Simulation Test—Aerobic Sewage Treatment—A: Activated Sludge Units; B: Biofilms, 2001. OECD Guidelines for the Testing of Chemicals, Section 3”, as was described by Zdarta et al. [38].

The making of all samples initially consisted of taking 4 mL of synthetic wastewater and diluting it with demineralized water, followed by sterilization (15 min, 1 atm, 123–126 °C). Ingredients were added to each sample according to the Table 1. The pH of the cultures was 5. For Culture 2 and 3, the inoculum was added after ozone treatment (mixed vigorously and left in a dark place for 24 h), and for Culture 4 and 5, the inoculum was added before the addition of creosote and ozonated water.

The ozonated water was prepared by ozone bubbling into demineralized water. The ozone was generated from oxygen in the BMT 802 N ozonator (BMT Messtechnik GMBH, Berlin, Germany). The ozone concentration in the gas stream in the inlet of the reactor was measured by a BMT 964BT ozone analyzer (BMT Messtechnik GMBH, Berlin, Germany). The ozone concentration in the ozonated water was determined using spectrophotometric measurements (with a Jasco V-630 apparatus).

#### 2.2.2. Biodegradation Tests

The five cultures in three replicates (15 incubation bottles) were incubated on a rotary shaker for 12 weeks at 30 °C in the dark. Samples were taken every two weeks for the analysis of residual hydrocarbons, total organic carbon, chemical oxygen demand, and microbial activity.

#### 2.2.3. Total Organic Carbon and Chemical Oxygen Demand

The total organic carbon (TOC) was determined using a two-stage process with the usage of a TOC-X5 shaker (HACH LANGE Sp. z o.o., Wrocław, Poland), a LT200 thermostat (HACH LANGE Sp. z o.o., Wrocław, Poland), a DR 3900 photometer (HACH LANGE Sp. z o.o., Wrocław, Poland) and LCK386 cuvettes (HACH LANGE Sp. z o.o., Wrocław, Poland ). In a two-stage process, the total inorganic carbon was first expelled with the help of the TOC-X5 shaker, and the TOC was then oxidized to carbon dioxide using the thermostat. The carbon dioxide passed through a membrane into the indicator cuvette, where it caused a color change to occur, and this was evaluated with a photometer. The detailed description of this method can be found in the working procedure [39]. The chemical oxygen demand (COD) was determined using a standard dichromate method with a LT200 thermostat (HACH LANGE Sp. z o.o., Ames, IA, USA), a DR 3900 photometer (HACH LANGE Sp. z o.o., Ames, IA, USA), and LCK314 cuvettes (HACH LANGE Sp. z o.o., Wrocław, Poland). A detailed description of this method can be found in the working procedure [40].

#### 2.2.4. Gas Chromatographic Analyses

For the quantitative and qualitative analysis of hydrocarbons, 30 mL of the cultures (mixed and shaken earlier to provide homogeneity of samples) were used. The samples were placed in 50 mL plastic tubes and extracted with 5 mL of hexane. They were then transferred to chromatographic vials and analyzed as follows: helium as a carrier gas (1 mL min^−1^); oven temperature program: 40 °C for the first 2 min and then increased to 300 °C at a 15 °C per min^−1^ rate (the final temperature was kept for 15 min). The analyses were conducted using a Pegasus 4D GCxGC-TOFMS (LECO, St. Joseph, MI, USA) equipped with a BPX-5 column (60 m, 250 μm, 0.25 μm). The obtained chromatograms are presented in the Supplementary Materials (Figures S1–S5). The quantity of the residual hydrocarbons was measured using a calibration curve, and the final content was corrected based on the values determined for the control and abiotic samples.

#### 2.2.5. Microbial Activity Measurements

Measurements of the bacteria cells’ activity were performed using a 3-(4,5-dimethylthiazol-2-yl)-2,5-diphenyltetrazolium bromide assay (MTT) according to the method described by [41]. Briefly, 0.5 mL of cultures were mixed with 0.05 mL of 5 g L^−1^ MTT solution and incubated for 48 h. After incubation, the cultures were centrifuged at 11,000× *g*. The supernatant was discarded, and the pellet (the formazan precipitate formed by viable cells) was dissolved with 0.25 mL of propane-2-ol. Afterward, the samples were centrifuged again at 4000× *g*, and the supernatant was analysed on a UV-VIS spectrophotometer at 560 nm.

#### 2.2.6. Statistical Analyses and Initial Reaction Rates

The results presented in the study were calculated as an average value from at least three independent experiments. A variance analysis and Student’s *t*-test were used to determine the statistical significance of differences between the average values. The differences were considered statistically significant at *p* < 0.05.

The initial reaction rates were calculated through the use of a differentiating exponential curve that fitted experimental points (concentration, time) at a correlation factor higher than 0.98.

The calculations were conducted using Excel 2019 (Microsoft Office Professional 2019) and OriginPro 2022 (OriginLab 2022) software.

## 3. Results

### 3.1. Creosote Hydrocarbons Degradation

**Figure 1:**
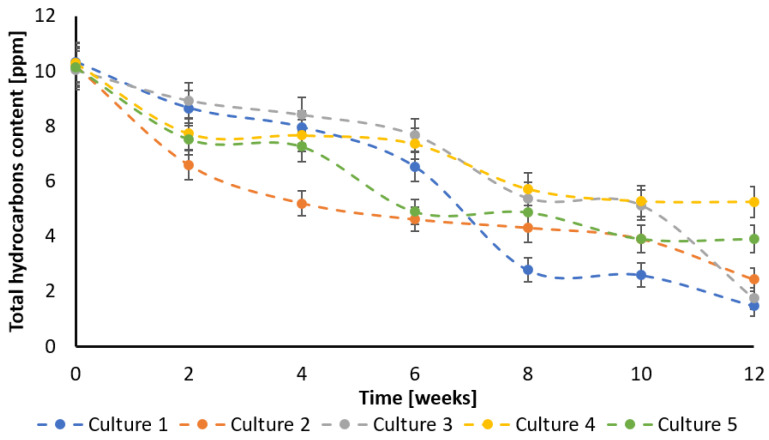
Changes in creosote concentration in different cultures.

The crucial parameter describing the self-cleaning potential of different tested systems was total hydrocarbon content (Figure 1). In Culture 1, without the ozonation process, the biodegradation was carried out relatively slowly during the first six weeks; when the hydrocarbons content dropped to 6.4 ppm, then degradation ratio increased, and after a further two weeks it reached 2.8 ppm, and then the process slowed down again. Finally, after 12 weeks, the creosote hydrocarbons content was 1.5 ppm. The cultures with pre-ozonation (No. 2 and 3) presented another process rate. The lower ozone concentration in Culture 2 promoted a more intensive biodegradation during the first weeks (reaching 5.2 ppm after four weeks) and then slowed down to reach a hydrocarbon concentration of 2.4 ppm at the end. Culture 3 showed the least biodegradation throughout the experiment, although the process accelerated significantly in the last two weeks. Finally, the hydrocarbon concentration in this Culture was 1.7 ppm, which was almost the same as in Culture 1. The cultures where the ozonation process was conducted after the inoculation with bacteria (No. 4 and 5) were characterized with relatively low biodegradation effectiveness. During the experiment, the rate of hydrocarbons removal was average, but they later appeared to be less effective than other cultures. After 12 weeks, the hydrocarbon concentrations were 5.3 ppm and 3.9 ppm for Culture 4 and 5, respectively.

The determined initial reaction rate of creosote decay equaled 0.00425 ± 0.000248, 0.01634 ± 0.00000163, 0.00181 ± 0.0000353, 0.0056 ± 0.000081 and 0.0109 ± 0.00017 ppm h^−1^ for cultures 1, 2, 3, 4, and 5, respectively. These results indicated that biodegradation using Culture 2 was most effective.

An additional perspective involved the monitoring of selected PAHs (Supplementary Materials, Figure S6), which were present in creosote oil. For the majority of the investigated hydrocarbons, Culture 2 appeared to be the most effective, especially in the first month of the experiment when the decrease in PAHs concentration was the most visible. The second best system was Culture 1, which was significantly less effective than Culture 2. Considering the biodegradability of the analyzed PAHs, the most resistant to degradation appeared to be quinoline, probably because of the higher toxicity caused by the presence of nitrogen in an aromatic ring. The less resistant to biodegradation were acenaphthylene and benz[a]anthracene, which were almost totally degraded after 12 weeks. However, it must be mentioned that the results refer to the primary biodegradation process, which indicates the decay of the initial form of the hydrocarbon molecule.

### 3.2. TOC and COD during Biodegradation

**Figure 2:**
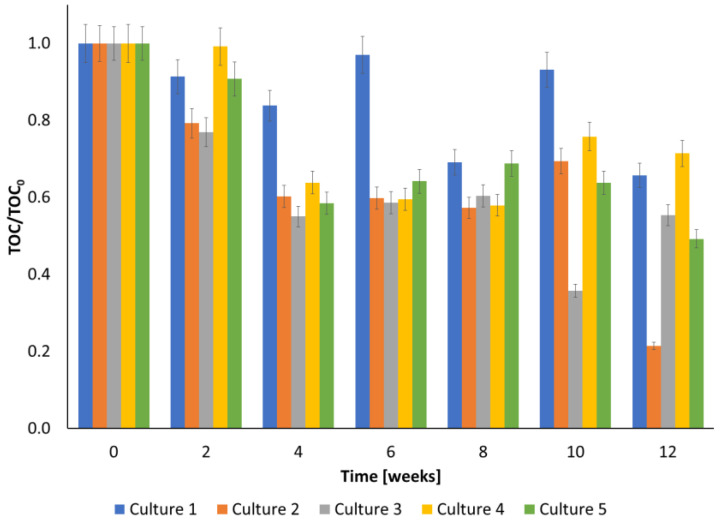
Changes in the TOC during the biodegradation process of creosote oil.

Figure 2 shows the changes in TOC during the biodegradation of creosote oil in different cultures. The decrease in TOC was observed for all cultures. The application of a higher ozone concentration (Cultures 3 and 5) caused a slightly greater TOC decrease in the first four weeks. The highest decay of this parameter after 12 weeks was observed for Culture 2 (almost 80% TOC was removed). It should be noted that the decrease of TOC in culture 1 was smaller than in other cultures, which proved that ozonation increased the efficiency of the biodegradation of creosote oil and its transformation products. It is likely caused by the transformation of PAHs into less toxic and more biodegradable compounds during ozonation.

**Figure 3:**
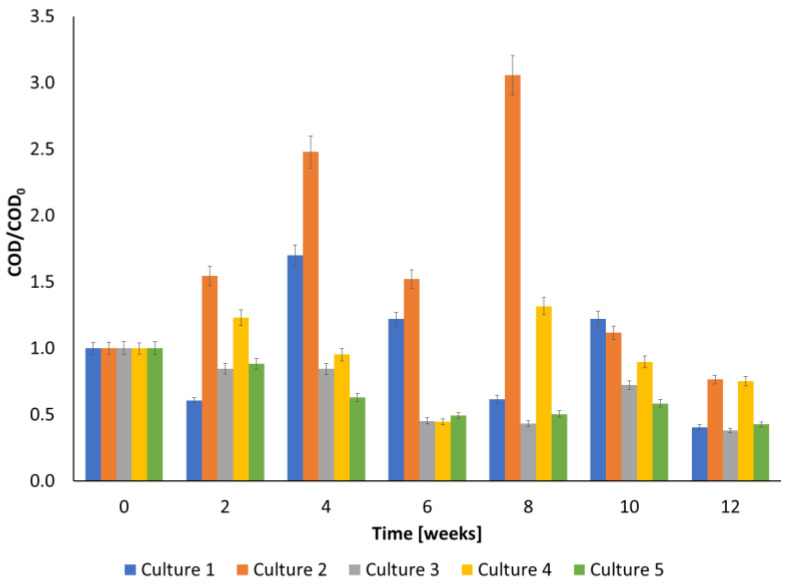
Changes in the COD during the biodegradation process of creosote oil.

The biodegradation was also compared in terms of COD reduction (Figure 3). In the case of the culture without the addition of ozone (Culture 1), after 2 weeks a decrease in this parameter was observed. After 4 weeks, the COD had increased, and after 12 weeks it had decreased by 60%. A significant increase of COD has been noted for Culture 2 during 8 weeks of biodegradation. The removal of COD after 12 weeks equaled 60, 23, 62, 25 and 57% for culture 1, 2, 3, 4 and 5, respectively.

### 3.3. Changes in Microbial Activity during Biodegradation

**Figure 4:**
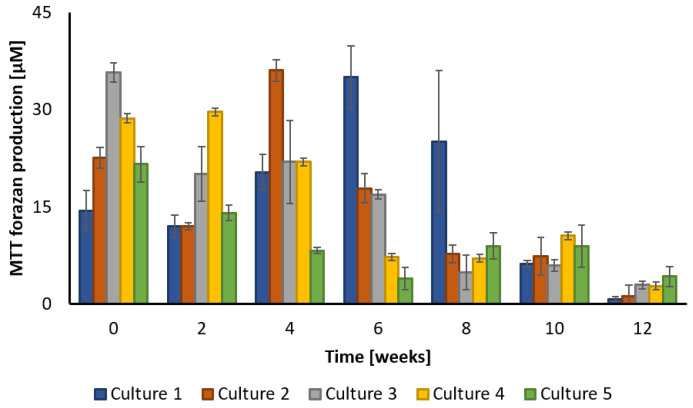
Microbial activity measured by MTT formazan production in different cultures.

Additional knowledge about the biodegradation process was provided thanks to the measurement of microbial activity in the cultures. The different cultures differ both in the values of cell metabolic activity and in the profile of changes in this parameter over time (Figure 4). In particular, this concerns the point at which cellular activity reaches its maximum. In the case of Culture 1, activity during the first weeks was stable and then rose to reach the maximum at 6 weeks, after which it declined steadily until the end. The cultures with pre-ozonation (Culture 2 and 3) were characterized with higher initial cell activity. The maximum values were measured at the beginning and after 4 weeks in case of Culture 3 and Culture 2, respectively. The maximum activity was reported at the start for Culture 5 as well. However, the absolute value was nearly 25% lower than for Culture 3. Culture 4 demonstrated stable activity during the first two weeks, after which it decreased gradually.

## 4. Discussion

The collected results draw attention to the complexity of the coupled ozonation-biodegradation process. The evaluation of the process effectiveness depends on the time perspective and parameter measured. Many studies describe results obtained after a shorter time, such as 12 or 28 days [42,43]. However, based on our previous studies on the biodegradation of creosote oil [6,8], we concluded that extending the time of the experiment would provide more promising results. This is because in earlier studies we observed that it took a relatively long time for microorganisms to adapt and that they had a relatively late entry into the logarithmic and stationary phases at the same time as they had a high level of multiplication of microorganisms. Thus, by observing the process for longer than a month, we can take into account the influence of microorganisms with slower growth rates but more efficient biodegradation, i.e., ultimately higher efficiency. Table 2 presents a comparison of the results obtained in this work with other studies. The ozone concentration applied in this work was much lower in comparison with the literature data, whereas the obtained reduction percentage of COD, total hydrocarbons, and individual PAHs were comparable or even higher than in other studies.

Considering PAHs disappearance in the cultures, pre-ozonation in small doses was the most successful approach, especially in first weeks of the experiment. However, the final concentration was comparable with that obtained in cultures with no pre-treatment. An analysis of the primary biodegradation of several PAHs monitored reveals analogical observations. The higher doses of pre-ozonation and ozonation after microbial inoculation appeared to be effective. The causes cannot be explained simply by the negative effect of ozonation on bacterial cells, because it is contradicted by measurements of cell activity. Rather, the time shift of the measured maximum cell metabolic activity indicates the prolonged bacterial adaption to the new conditions. Moreover, the high reduction of COD in cultures with high oxidation doses and biodegradation only bring an additional perspective.

The highest decay of TOC was achieved in the culture that was pre-ozonated with small ozone doses. According to the research performed by Chen et al., petroleum hydrocarbons can be effectively removed from soils by using sequential biodegradation and ozonation [23]. The application of this combined method made it possible to achieve the 40–45% removal of TOC [23] and to meet the regulatory standard for this parameter, while biodegradation alone was unable to meet the standard. Moreover, dissolved organic carbon was the dominant substrate for microorganisms when readily biodegradable hydrocarbons were no longer available. In the case of relatively biodegradable petroleum hydrocarbons, pre-ozonation and post-ozonation strategies were equally effective, while post-ozonation was more efficient for the less biodegradable hydrocarbons [23].

Ozonation can improve the bioavailability and biodegradability of contaminants through the oxidation of organics with unsaturated functional groups. Ozone in water solutions is decomposed into oxygen relatively quickly. The ozone decay rate is mainly influenced by the folowing parameters: temperature, pH, as well as the presence of organic and inorganic compounds and other medium components. The half-life of ozone in distilled water at ambient temperature is approximately 25 s at pH 10, 17 min at pH 7, and 7 h at pH 4 [44]. The ozone concentration at the beginning of the biodegradation tests equaled to 0.76 ± 0.17 and 6.63 ± 0.68 mg L^−1^, respectively (Table 1). Taking into account the parameters of the experiments, the pH of the cultures that equaled 5, and presence of organic compounds, it can be assumed that the ozone was completely consumed after about 4 h of the reaction. It is commonly known that many bacterial strains are very sensitive to ozone action, therefore it is used in disinfection processes. It is worthy of note that ozone is also consumed in the decomposition of other medium components, such as PAHs. In cases when the reaction of ozone with chemicals proceeds very rapidly, the adverse effect of ozone on bacteria is hardly visible. The obtained results showed that cultures with the addition of ozone were characterized by higher initial cell activity in comparison with the culture without ozone addition (Figure 4). However, this parameter was lower in cases of the addition of bacteria soon after water was ozonated with a higher ozone concentration (Culture 5) compared to those where a lower ozone concentration was used (Culture 4). Thus, the concentration of ozone is an important factor that influenced this adverse effect on the bacteria. According to the literature reduction rate of *Pseudomonas aeruginosa* cells increased with the amount of transferred ozone dose increasing from 11 to 45 mg L^−1^ [45]. Ozonation with an ozone dose of 5 and 10 mg L^−1^ was not able to eliminate *Pseudomonas* from the secondary effluents of two wastewater treatment plants [46].

The performed studies showed that bacterial activity reached its maximum and then decreased. This suggests that in the cultures there are no more organic compounds that could be degraded, or that there could be some toxic products which have an adverse effect on the bacteria. The brief ozonation of pyrene significantly decreases the toxicity of its intermediates, as evidenced by the increased biological oxygen demand measured in the effluent and a decrease in *E. coli* inhibition [47]. The degradation of pyrene can be initiated by O_3_ via ring cleavage, and further oxidation ensued via reactions with both ozone (direct ozonation) and hydroxyl radicals (indirect ozonation) until complete mineralization was reached [45]. According to the research performed by Yang et al., the toxicity of phenanthrene byproducts formed during ozonation was also lower than for that of the parent compound [48]. Microbubble ozonation completely removed the acute toxicity of benzo[a]pyrene to *Daphnia magna*, whereas the toxicity reduction by macrobubble ozonation was not consistent, owing possibly to toxic degradation products [42]. Cui et al. [49] investigated the coking wastewater, which contains high concentrations of cyanide, phenols, pyridine, quinoline, and polycyclic aromatic hydrocarbons. The toxicity of this wastewater effluent in cases of the application of the simultaneous combination of ozonation and biodegradation was 327% and 306% lower than that of the individual biodegradation and ozontion system, respectively [49]. In the case of the ozonation system, the toxicity fluctuated slightly and presented an increasing trend, indicating that more toxic intermediates were produced [49].The use of ozone treatment as a step to aid the biodegradation of persistent organic pollutants has already been tested in the case of pharmaceutics, such as with tetracycline [50] or citalopram [51]. In the referenced articles, a reduced toxicity of the pollutants was observed; however, the authors noted the thread of transformation products. The ozonation-biodegradation was applied to urban wastewater [33] as well. In ozonation-biodegradation, in addition to the pollutants content, the concentration of soluble organic matter also affects the effectiveness of the process [52]. The studies performed by Bernal-Martinez et al. showed that ozonation pre-treatment of sludge increased the biodegradability or bioavailability of each PAH, and the PAH removals are correlated to their solubility [34]. The extended polycondensation of benzene rings in polyaromatic pollutants confers them with high chemical stability and low water solubility which limits their bioavailability and removal rates. Therefore, low-molecular weight compounds can be degraded faster than the highest ones [35].

One of the crucial factors that can be important in understanding the results was the application of bacterial strains that were primarily isolated from hydrocarbon-contaminated soils. They were used to degrade PAHs, but they had to adapt to degrade PAHs ozonation products. It is not uncommon for PAHs and their metabolites to affect the degradation of PAHs. According to Zhang et al. [43], the co-metabolism of microorganisms enables the utilization of poorly available PAHs in the presence of readily available ones that provide a source of carbon and energy. For example, *Micrococcus* sp. did not degrade anthracene, pyrene, or fluoranthene before naphthalene and phenanthrene were added, which increased the degradation of all PAHs tested. Furthermore, replacing the monoculture with co-cultures increases the bioavailability of contaminants due to microbial enzymes induced by readily available contaminants [4]. In this study, both the presence of several bacterial strains and the presence of 10 different PAHs undoubtedly enhanced the biodegradation of PAHs in wastewater. Special attention should be paid to the superior degradation of PAHs with more benzene rings (e.g., acenaphthylene, phenanthrene, pyrene, and benz[a]anthracene).

**Table 2:** Comparison of COD reduction and PAHs removal during biodegradation and ozonation.

Medium	Time of Study	C_O3_	COD Reduction (bio + O_3_)	Microorganisms	BTP Reduction	PYR Reduction	PHE Reduction	FLU Reduction	THC Reduction	Ref.
coking wastewater	12 h	30 mg L^−1^ h^−1^	48.5%	*Comamonadaceae*, *Paracoccus*, *Comamonas*, *Corynebacterium*, *Truepera microbes*	-	-	-	-	-	[49]
soil	9 weeks	2 g/h	-	-	~61% after 7 days bio + 3 h O_3_	~66% after 7 days bio + 3 h O_3_	-	-	~100%	[35]
soil	bio: 4 weeks, O_3_: 2 days	12 mg/day	-	-	23.2% (bio + O_3_); 73.5% (O_3_ + bio)	10.5% (bio + O_3_); 40.8% (O_3_ + bio)	46.5% (bio + O_3_); 71.6% (O_3_ + bio)	~100%	-	[42]
soil	12 days	20–790 mg L^−1^	-	*Pseudomonas* sp.	-	-	-	-	90%	[53]
simulated creosote wastewater	12 weeks	O_3_1: 0.76; O_3_2: 6.63 mg L^−1^	23.4% (O_3_1 + bio); 62.0% (O_3_2 + bio); 24.8% (bio + O_3_1); 57.3% (bio + O_3_2)	*Pseudomonas* sp. MChB, *Pseudomonas* sp. OS4, *Raoultella planticola* SA2, *Achromobacter* sp. KW1, *Rahnella aquatilis* DA2	83% (bio); 89% (O_3_1 + bio); 59% (O_3_2 + bio); 43% (bio + O_3_1); 51% (bio + O_3_2)	89% (bio); 91% (O_3_1 + bio); 76% (O_3_2 + bio); 92% (bio + O_3_1); 87% (bio+ O_3_2)	96% (bio); 97% (O_3_1 + bio); 43% (O_3_2 + bio); 86% (bio + O_3_1); 36% (bio + O_3_2)	82% (bio), 88% (O_3_1 + bio); 62% (O_3_2 + bio); 43% (bio + O_3_1); 52% (bio+ O_3_2)	86% (bio), 76% (O_3_1 + bio); 83% (O_3_2 + bio); 49% (bio + O_3_1); 61% (bio+ O_3_2)	this work

Bio—biodegradation, O_3_—ozonation, C_O3_—concentration of ozone, O_3_1—ozone concentration equal to 0.76 mg L^−1^, O_3_2—ozone concentration equal to 6.63 mg L^−1^, PYR—pyrene, PHE—phenanthrene, FLU—fluorene, BTP—benzo[b]thiophene, THC—total hydrocarbons.

## 5. Conclusions

This study presents the results of PAHs degradation using an ozonation-biodegradation hybrid system. The performed experiments showed that pre-ozonation increased the efficiency of the biodegradation of creosote oil. The combination of biodegradation and ozone pre-treatment using a small dose of ozone was the most effective in PAHs removal, especially in the first weeks of the biodegradation experiment. However, the final concentration was comparable with that obtained in culture with no pre-treatment. The determined initial reaction rate of creosote decay equaled 0.00425 ± 0.000248, 0.01634 ± 0.00000163, 0.00181 ± 0.0000353, 0.0056 ± 0.000081 and 0.0109 ± 0.00017 ppm h^−1^ for Cultures 1, 2, 3, 4, and 5, respectively. The highest decay of TOC after 12 weeks was observed for the culture pre-ozonated using a small dose of ozone (almost 80% TOC was removed). The removal of COD after 12 weeks equaled 60, 23, 62, 25 and 57%, for Cultures 1, 2, 3, 4, and 5, respectively. Quinoline appeared to be most resistant to degradation. Less resistant to biodegradation were acenaphthylene and benz[a]anthracene, which were degraded almost totally after 12 weeks. The applied cultures differ both in the values of cell metabolic activity and in the profile of changes in this parameter over time. The maximum cellular activity was reached at 6, 4, 0, 2 and 0 weeks of biodegradation for Cultures 1, 2, 3, 4, and 5, respectively.

## Figures and Tables

**Table 1:** Procedure of culture preparation.

Culture	Concentration of Ozone, mg L^−1^	Volume of Synthetic Wastewater, mL	Volume of Bacteria Inoculum before Adding Ozone, mL	Volume of Creosote, mL	Volume of Ozonated Water Addition, mL	Volume of Bacteria Inoculum after Adding Ozone, mL	Final Volume, mL
1	-	4	20	0.4	-	-	420.4
2	0.76 ± 0.17	4	-	0.4	20	20	420.4
3	6.63 ± 0.68	4	-	0.4	200	20	420.4
4	0.76 ± 0.17	4	20	0.4	20	-	420.4
5	6.63 ± 0.68	4	20	0.4	200	-	420.4

## Data Availability

Not applicable.

## Supplementary Materials

The following supporting information can be downloaded at: https://www.mdpi.com/article/10.3390/ijerph20075347/s1, Figure S1: GC spectrum of the culture 1 during the biodegradation process; AZU—azulene, BTP—benzo[b]thiophene, QUI—quinoline, ENP—1-ethylnaphthalene, ANP—acenaphthylene, ANT—anthracene, FLU—fluorene, PHE—phenanthrene, PYR—pyrene; Figure S2: GC spectrum of the culture 2 during the biodegradation process; AZU—azulene, BTP—benzo[b]thiophene, QUI—quinoline, ENP—1-ethylnaphthalene, ANP—acenaphthylene, ANT—anthracene, FLU—fluorene, PHE—phenanthrene, PYR—pyrene; Figure S3: GC spectrum of the culture 3 during the biodegradation process; AZU—azulene, BTP—benzo[b]thiophene, QUI—quinoline, ENP—1-ethylnaphthalene, ANP—acenaphthylene, ANT—anthracene, FLU—fluorene, PHE—phenanthrene, PYR—pyrene; Figure S4: GC spectrum of the culture 4 during the biodegradation process; AZU—azulene, BTP—benzo[b]thiophene, QUI—quinoline, ENP—1-ethylnaphthalene, ANP—acenaphthylene, ANT—anthracene, FLU—fluorene, PHE—phenanthrene, PYR—pyrene; Figure S5: GC spectrum of the culture 5 during the biodegradation process; AZU—azulene, BTP—benzo[b]thiophene, QUI—quinoline, ENP—1-ethylnaphthalene, ANP—acenaphthylene, ANT—anthracene, FLU—fluorene, PHE—phenanthrene, PYR—pyrene; Figure S6: Degradation of selected hydrocarbons: (A) azulene, (B) benzo[b]thiophene, (C) quinoline, (D) 1-ethylnaphthalene, (E) acenaphthylene, (F) anthracene, (G) fluorene, (H) phenanthrene, (I) pyrene, (J) benz[a]anthracene.
