# Supplementary material for: Application of Ozonation-Biodegradation Hybrid System for Polycyclic Aromatic Hydrocarbons Degradation
Source: Int J Environ Res Public Health. 2023 Mar 31;20(7):5347. doi: 10.3390/ijerph20075347 (PMC10094057; doi:10.3390/ijerph20075347)
Supplement: Supplementary file 1 [file ijerph-20-05347-s001.zip › ijerph-2288760-supplementary.pdf]

Supplementary Materials

Application of Ozonation-Biodegradation Hybrid System for Polycyclic Aromatic Hydrocarbons Degradation

Magdalena Olak-Kucharczyk [1,*], Natalia Festinger [1] and Wojciech Smułek [2]

[1] Łukasiewicz Research Network – Lodz Institute of Technology, Maria Skłodowska-Curie 19/27, 90-570 Łódź, Poland
[2] Institute of Chemical Technology and Engineering, Poznan University of Technology, Berdychowo 4, 60-695 Poznan, Poland
* Corresponding author: magdalena.olak-kucharczyk@lit.lukasiewicz.gov.pl

Figure S1 GC spectrum of the culture 1 during the biodegradation process; AZU – azulene, BTP – benzo[b]thiophene, QUI – quinoline, ENP – 1-ethylnaphthalene, ANP – acenaphthylene, ANT – anthracene, FLU – fluorene, PHE – phenanthrene, PYR – pyrene;
Figure S2 GC spectrum of the culture 2 during the biodegradation process; AZU – azulene, BTP – benzo[b]thiophene, QUI – quinoline, ENP – 1-ethylnaphthalene, ANP – acenaphthylene, ANT – anthracene, FLU – fluorene, PHE – phenanthrene, PYR – pyrene;
Figure S3 GC spectrum of the culture 3 during the biodegradation process; AZU – azulene, BTP – benzo[b]thiophene, QUI – quinoline, ENP – 1-ethylnaphthalene, ANP – acenaphthylene, ANT – anthracene, FLU – fluorene, PHE – phenanthrene, PYR – pyrene;
Figure S4 GC spectrum of the culture 4 during the biodegradation process; AZU – azulene, BTP – benzo[b]thiophene, QUI – quinoline, ENP – 1-ethylnaphthalene, ANP – acenaphthylene, ANT – anthracene, FLU – fluorene, PHE – phenanthrene, PYR – pyrene;
Figure S5 GC spectrum of the culture 5 during the biodegradation process; AZU – azulene, BTP – benzo[b]thiophene, QUI – quinoline, ENP – 1-ethylnaphthalene, ANP – acenaphthylene, ANT – anthracene, FLU – fluorene, PHE – phenanthrene, PYR – pyrene;
Figure S6 Degradation of selected hydrocarbons: a) azulene, b) benzo[b]thiophene, c) quinoline, d) 1-ethylnaphthalene, e) acenaphthylene, f) anthracene, g) fluorene, h) phenanthrene, i) pyrene, j) benz[a]anthracene;

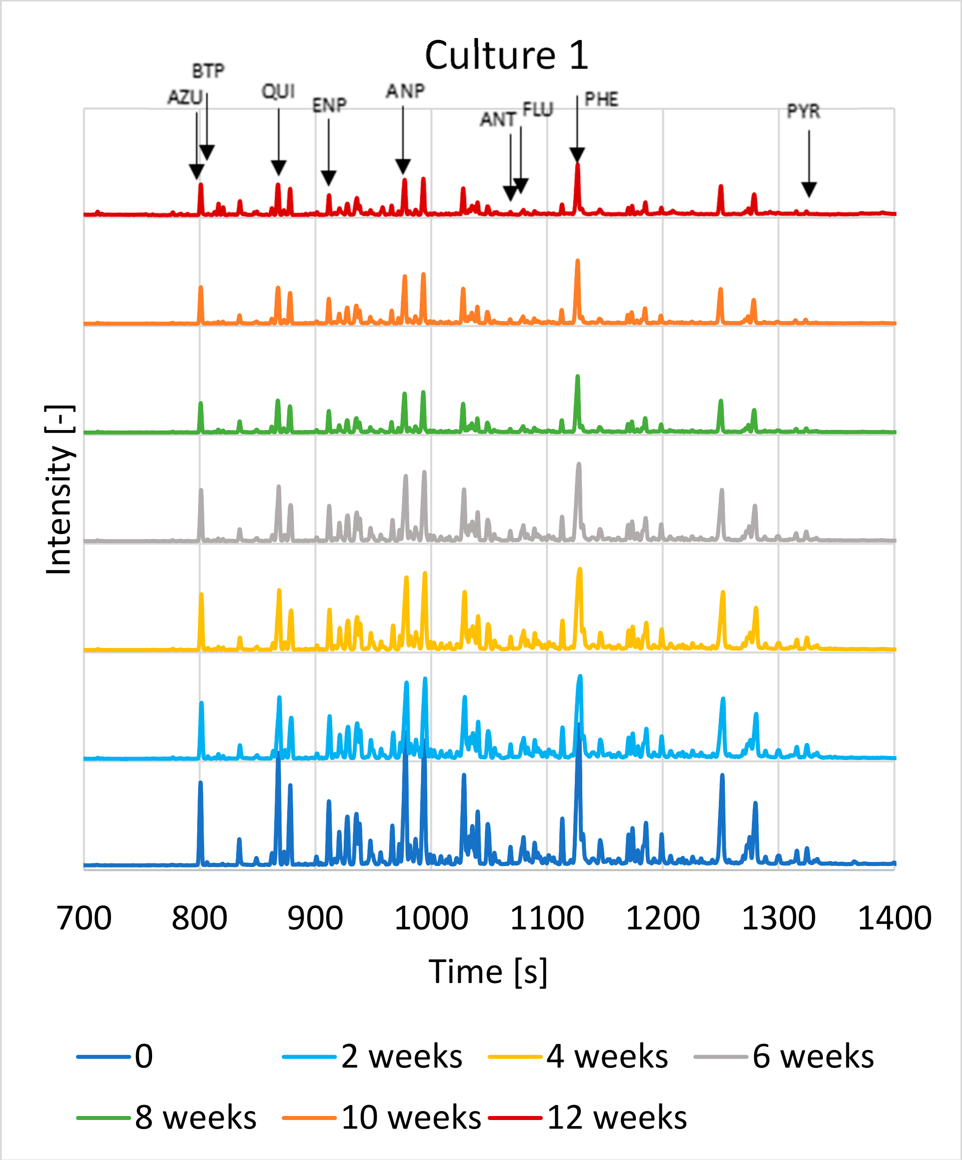

Figure S1. GC spectrum of the culture 1 during the biodegradation process; AZU – azulene, BTP – benzo[b]thiophene, QUI – quinoline, ENP – 1-ethylnaphthalene, ANP – acenaphthylene, ANT – anthracene, FLU – fluorene, PHE – phenanthrene, PYR – pyrene.

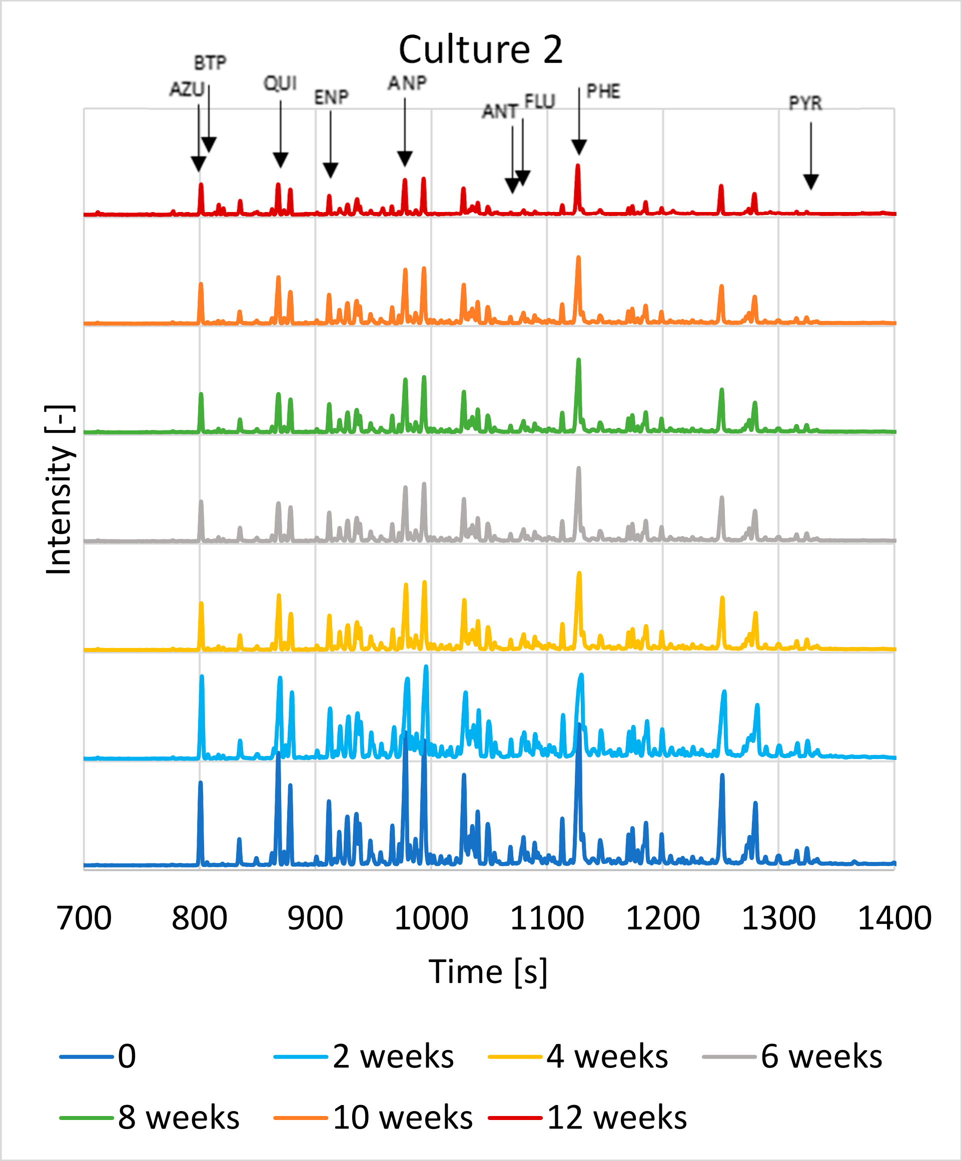

Figure S2. GC spectrum of the culture 2 during the biodegradation process; AZU – azulene, BTP – benzo[b]thiophene, QUI – quinoline, ENP – 1-ethylnaphthalene, ANP – acenaphthylene, ANT – anthracene, FLU – fluorene, PHE – phenanthrene, PYR – pyrene.

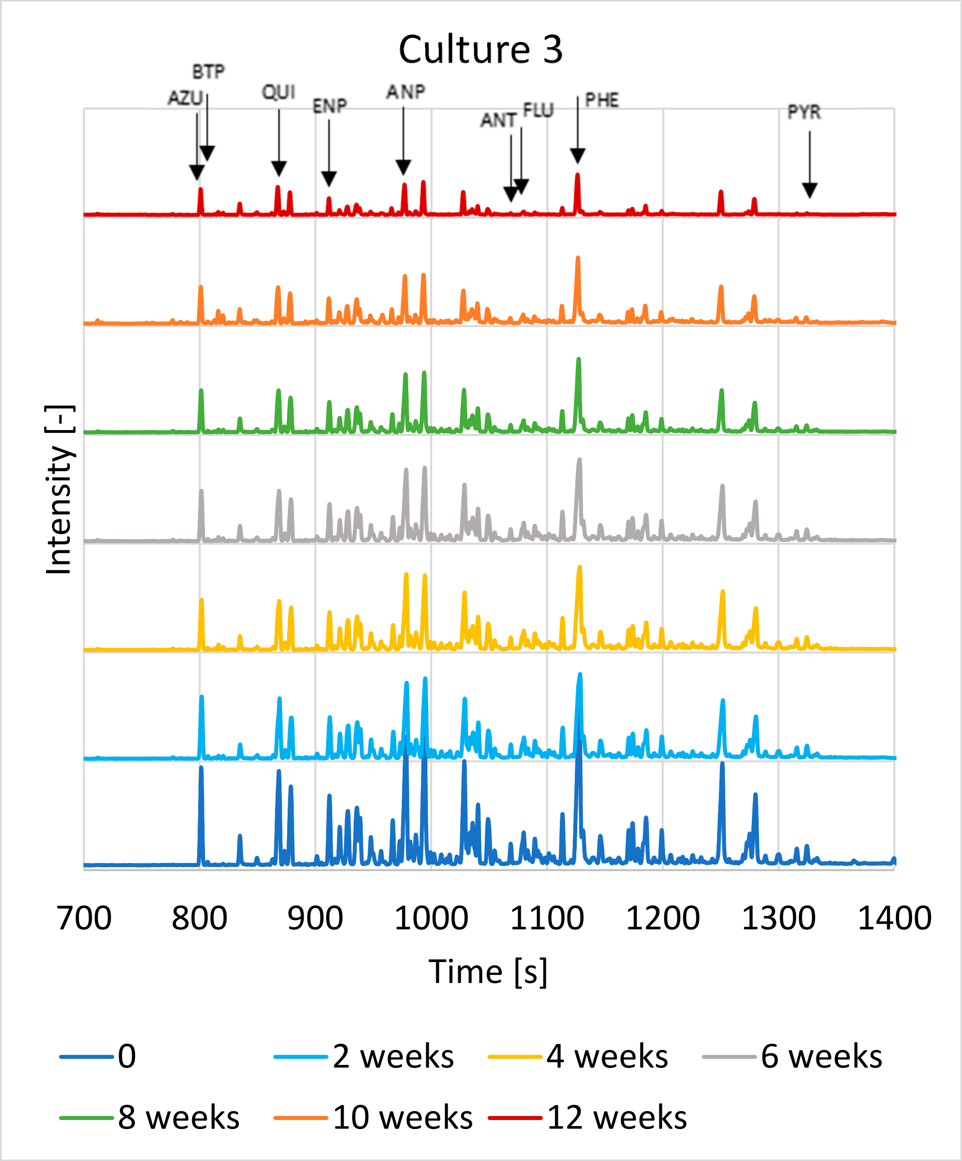

Figure S3. GC spectrum of the culture 3 during the biodegradation process; AZU – azulene, BTP – benzo[b]thiophene, QUI – quinoline, ENP – 1-ethylnaphthalene, ANP – acenaphthylene, ANT – anthracene, FLU – fluorene, PHE – phenanthrene, PYR – pyrene.

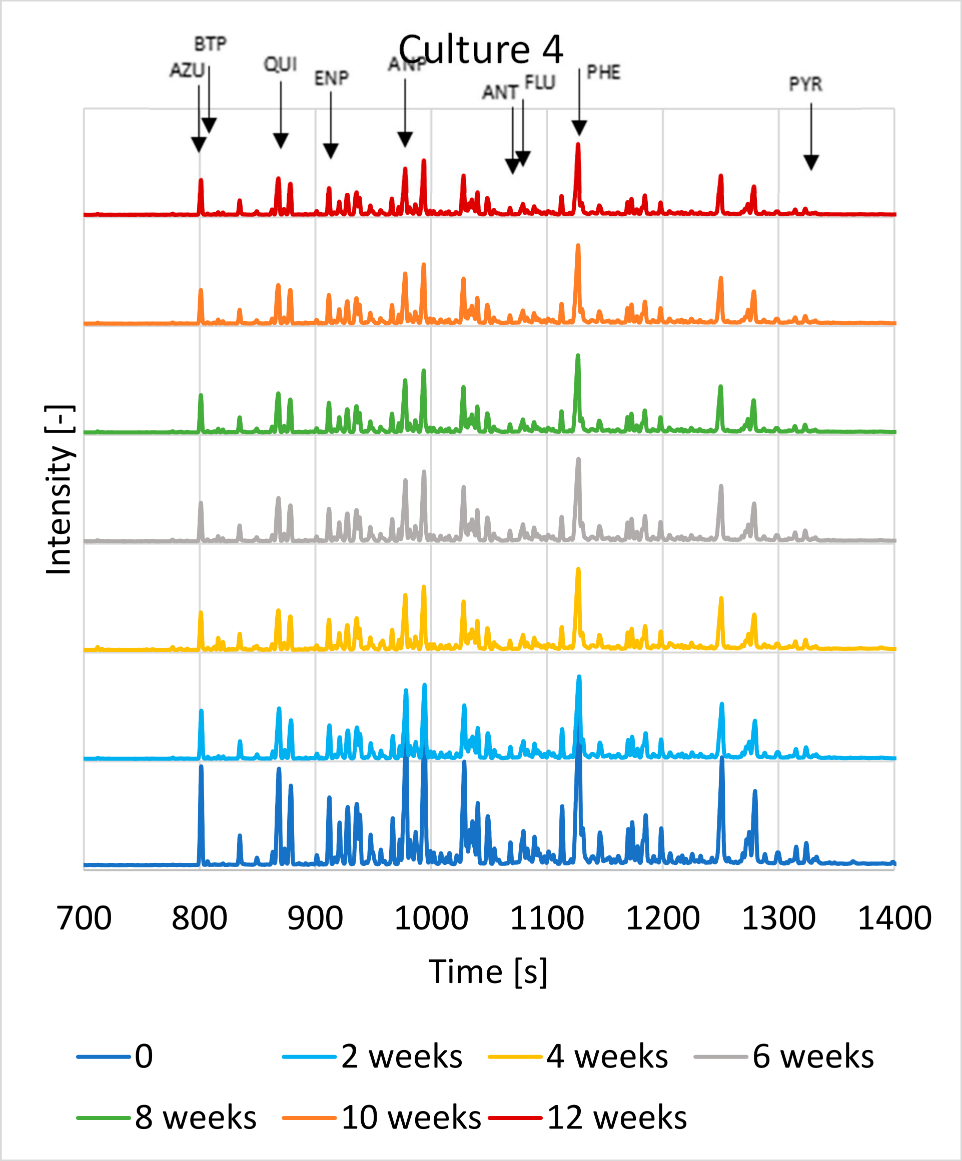

Figure S4. GC spectrum of the culture 4 during the biodegradation process; AZU – azulene, BTP – benzo[b]thiophene, QUI – quinoline, ENP – 1-ethylnaphthalene, ANP – acenaphthylene, ANT – anthracene, FLU – fluorene, PHE – phenanthrene, PYR – pyrene.

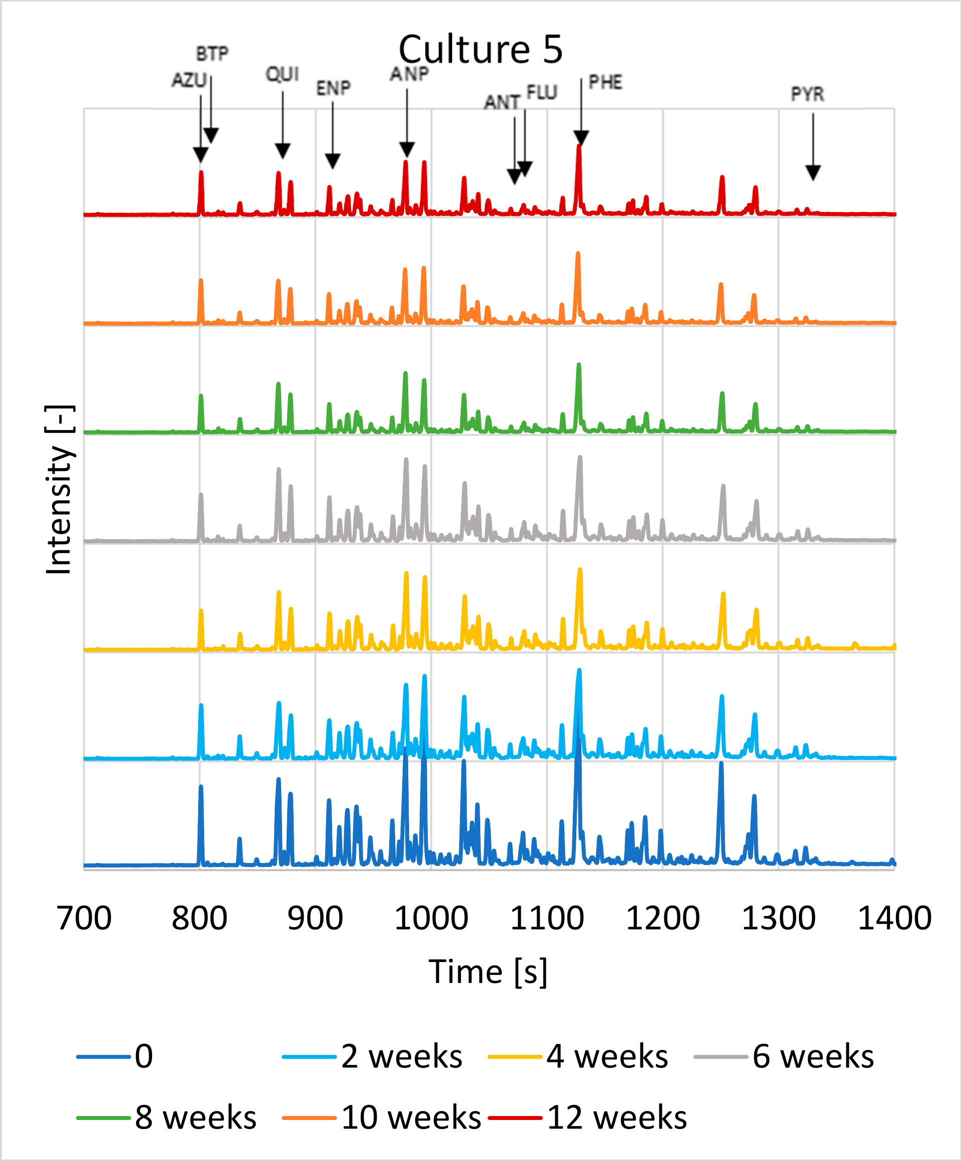

Figure S5. GC spectrum of the culture 5 during the biodegradation process; AZU – azulene, BTP – benzo[b]thiophene, QUI – quinoline, ENP – 1-ethylnaphthalene, ANP – acenaphthylene, ANT – anthracene, FLU – fluorene, PHE – phenanthrene, PYR – pyrene.

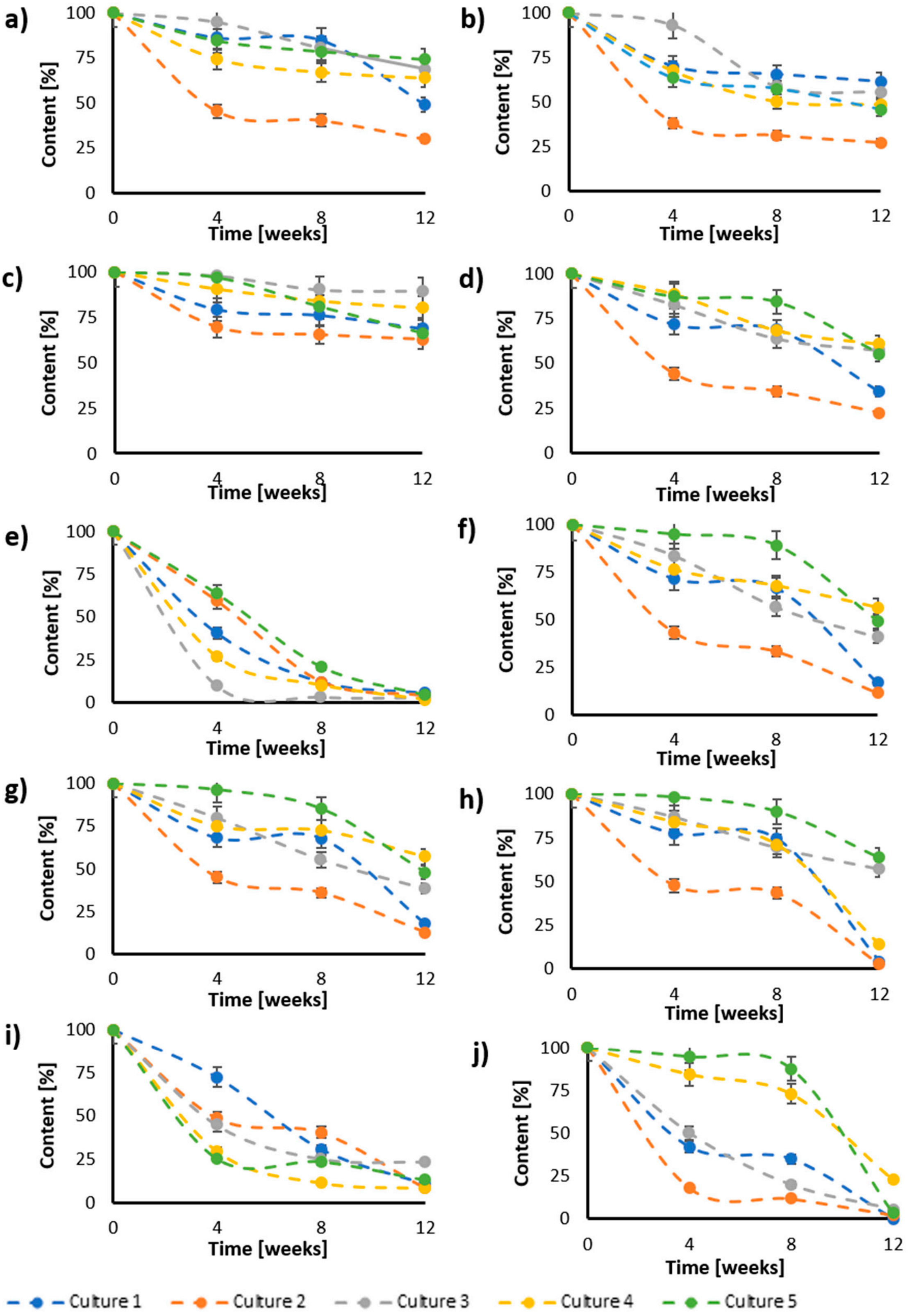

Figure S6. Degradation of selected hydrocarbons: a) azulene, b) benzo[b]thiophene, c) quinoline, d) 1-ethylnaphthalene, e) acenaphthylene, f) anthracene, g) fluorene, h) phenanthrene, i) pyrene, j) benz[a]anthracene.
